# Several key issues must be noted in determining postoperative analgesic efficacy of intercostal nerve block for thoracoscopic surgery

**DOI:** 10.1186/s13019-023-02456-2

**Published:** 2023-12-01

**Authors:** Xin-Tao Li, Wen-He Yang, Fu-Shan Xue

**Affiliations:** Department of Anesthesiology, Beijing Friendship Hospital, Capital Medical University, No. 95 Yong-An Road, Xi-Cheng District, Beijing, 100050 People’s Republic of China

**Keywords:** Postoperative pain, Intercostal nerve block, Thoracoscopic surgery, Enhanced recovery after surgery

## Abstract

The letter to the editor was written in response to “The effect of ultrasound-guided intercostal nerve block on postoperative analgesia in thoracoscopic surgery: a randomized, double-blinded, clinical trial”, which was recently published by Li et al. (J Cardiothorac Surg 18(1):128, 2023). In this article, Li et al. showed that addition of a preoperative intercostal nerve block to the multimodal analgesic strategy significantly reduced the pain scores within 48 h after surgery. However, we noted several issues in this study that were not well addressed. They were no use of a standard opioid-sparing multimodal analgesic strategy recommended in the current Enhanced Recovery After Surgery protocols for thoracic surgery, the lack of clear description for reasonable selection of rescue analgesics, the interpretion of between-group differences in the postoperative pain scores based on only statistical differences rather than clinically meaningful differences, inclusion of patients who were not blinded to study intervention, not reporting cumulative opioid consumption and complications of intercostal nerve block. We believe that clarification of these issues is not only useful for improving design quality of randomized clinical trials which assess postoperative analgesic efficacy of nerve blocks, but also is helpful for the readers who want to use an opioid-sparing multimodal protocol including a nerve block in patients undergoing thoracoscopic surgery.

## To the Editor:

By a randomized, double-blinded, clinical trial of 119 patients undergoing thoracoscopic pulmonary resection, Li et al. [1] determined the effects of adding a preoperative ultrasound-guided intercostal nerve block (ICNB) to the multimodal analgesic strategy on postoperative pain control and showed that the addition of an ICNB significantly reduced the visual analog scale (VAS) pain scores within 48 h after surgery. In addition to the limitations described by the authors in discussion section, however, there are several issues in the methods and results of this study which deserve further clarification and discussion.

First, this study actually used an opioid-based multimodal analgesic strategy, i.e., patient controlled analgesia (PCA) with intravenous sufentanil. This does not meet the requirements of the standard opioid-sparing multimodal analgesic strategy recommended in the current Enhanced Recovery After Surgery (ERAS) protocols for thoracic surgery, in which other than a nerve or fascial plane block, a serial of non-opioid basic analgesics with different mechanisms, such as paracetamol, nonsteroidal anti-inflammatory drugs or cyclooxygenase-2 specific inhibitors, gabapentin, dexamethasone, are included in order to achieve adequate co-analgesia and decrease opioid consumption. Most important, it is required that application of these non-opioid analgesics should be started before or during operation and regularly repeated after surgery, and opioids should be administered as rescue analgesia only when non-opioid basic analgesics are ineffective or contraindicated [2]. A prospective randomized control trial in patients undergoing thoracoscopic pulmonary resection demonstrated that the addition of a fascial plane block to the standard opioid-sparing multimodal analgesia did not provide any benefit in term of postoperative recovery quality, opioid consumptions and overall pain control [3]. Thus, we argue that different results regarding effects of adding a preoperative ICNB to the multimodal analgesic strategy on postoperative pain control for patients undergoing thoracic surgery would have been obtained, if a standard opioid-sparing multimodal analgesia strategy had been included in this study design. Furthermore, this design issue would also limit generalization of their findings into the current ERAS practice for thoracic surgery.

Second, parecoxib sodium was intravenously given for rescue analgesia when postoperative rest pain VAS score in the ward was > 3. Furthermore, the need of rescue analgesia during 48 h postoperatively was significantly decreased in the patients receiving the ICNB compared with control patients. In fact, parecoxib sodium only is a weak basic analgesic. As a main goal of ERAS programs is to minimize opioid use, it may be better to use parecoxib sodium as a rescue analgesic for moderate pain. However, opioids should still be standard rescue analgesics for inadequate postoperative pain control with non-opioid basic analgesic [4]. In fact, the PCA with intravenous sufentanil used in this study also included the bolus doses for inadequate postoperative pain control. We would like to know whether the bolus doses of PCA were included in the need of rescue analgesia and the cumulative sufentanil consumptions with PCA were comparable between groups. Without these data, we believe that comparing the needs of rescue analgesia during 48 h postoperatively between groups is inappropriate.

Third, pain VAS score at each observed point within 48 h postoperatively was statistically significantly lower in the patients receiving the ICNB compared to the control patients. Other than 0 and 4 h postoperatively, however, means of pain VAS scores at other time points postoperatively were 3 or less, indicating that most patients in the two groups only experienced mild postoperative pain, which achieves postoperative pain control goal of ERAS programs for thoracic surgery [2]. Other than 0 h time point, moreover, the net between-group differences in the means of rest pain VAS scores at other observed points were also less than 1, which is smaller than the desired minimum difference between groups in this study design. Furthermore, available literature recommends that the minimal clinically important difference of postoperative pain score required in a randomized controlled trial comparing different interventions is 1.5 when pain levels are assessed by a 0–10 VAS [5]. That is, the improvement of postoperative pain control by adding a single-injection ICNB to the multimodal analgesia does not exceed the desired minimum difference between groups in this study design or the recommended minimal clinically important difference in available literature. In these cases, we cannot determine if a brief and slight improvement of early postoperative pain control by adding a single-injection ICNB to the multimodal analgesia should be considered as being clinically important.

Finally, this study showed that duration of chest tube insertion, an outcome variable of the ERAS programs for thoracic surgery [2], was significantly reduced by addition of single-injection ICNB. However, we found that other important outcome variables of the ERAS programs for thoracic surgery, such as postoperative hospital stay, incidence of postoperative nausea and vomiting and the occurrence of pulmonary infection [2], were not statistically different between groups. Furthermore, there were other issues that were not well addressed in this study, i.e., patient were not blinded to the study intervention, actual measures of this study did not include the cumulative opioid consumptions and hypothetical decrease in the cumulative opioid consumption in the study group, and there was not any reported complications from the single-injection ICNB. In addition, performance of ultrasound-guided ICNB also was a skillset that required moderately experienced practitioner. Thus, we consider that real values of adding a single-injection ICNB to the multimodal analgesia in the current ERAS practice for thoracic surgery need further verification.

## Data Availability

Not applicable.

## Abbreviations

ICNBP: Intercostal nerve block
PCA: Patient controlled analgesia
VAS: Visual analog scale
ERAS: Enhanced recovery after surgery

## References

[1] Li S, Feng J, Fan K, Fan X, Cao S, Zhang G (2023). The effect of ultrasound-guided intercostal nerve block on postoperative analgesia in thoracoscopic surgery: a randomized, double-blinded, clinical trial. J Cardiothorac Surg.

[2] Semenkovich TR, Hudson JL, Subramanian M, Kozower BD (2018). Enhanced recovery after surgery (ERAS) in thoracic surgery. Semin Thorac Cardiovasc Surg.

[3] Klaibert B, Lohser J, Tang R, Jew M, McGuire A, Wilson J (2022). Efficacy of ultrasound-guided single-injection erector spinae plane block for thoracoscopic wedge resection: a prospective randomized control trial. Reg Anesth Pain Med.

[4] Thompson C, French DG, Costache I (2018). Pain management within an enhanced recovery program after thoracic surgery. J Thorac Dis.

[5] Doleman B, Leonardi-Bee J, Heinink TP, Boyd-Carson H, Carrick L, Mandalia R, Lund JN, Williams JP (2021). Pre-emptive and preventive NSAIDs for postoperative pain in adults undergoing all types of surgery. Cochrane Database Syst Rev.

